# Antimicrobial resistance patterns of *Klebsiella pneumoniae* clinical isolates in the Qassim region, Saudi Arabia: a surveillance study (2015–2023)

**DOI:** 10.1186/s12879-026-14159-9

**Published:** 2026-08-01

**Authors:** Noorah Al-Sowayan, Farah Al-masoud, Manal Almutari, Malak Almunife, Tahani Esmail

**Affiliations:** 1 Department of Biology, College of Science, Qassim University, Buraydah, Saudi Arabia; 2 Department of Medical Laboratory, College of Applied Medical Sciences, Qassim University, Buraydah, Saudi Arabia; 3 Laboratory Blood Bank Administration Buraydah, Buraydah, Saudi Arabia; 4 King Fahad Specialist Hospital, Buraydah, Saudi Arabia; 5 Department of Statistics and Operations Research, College of Science, Qassim University, Buraydah, 51482 Saudi Arabia

**Keywords:** *K. pneumoniae*, Antibiotic resistance, ESBL, MDR, Nosocomial infections, Al-Qassim

## Abstract

**Background:**

Antibiotic resistance in *Klebsiella pneumoniae* is a major threat to global health. Despite its clinical importance, regional data from Saudi Arabia remain limited. This study analyzed the resistance patterns of *K. pneumoniae* isolates collected from the King Fahad Specialist Hospital in Al-Qassim between January 2015 and December 2023.

**Methodology:**

A retrospective cross-sectional analysis was conducted on 3,838 confirmed *K. pneumoniae* cases. Data were extracted from hospital electronic records and analyzed using R software. Descriptive and inferential statistics were used to examine resistance patterns by specimen type, age, sex, and hospital department.

**Results:**

High resistance rates were observed to first-line antibiotics, particularly third-generation cephalosporins (59.18%–68.69%), amoxicillin-clavulanate (58.38%), and piperacillin-tazobactam (52.81%). Resistance to fluoroquinolones ranged from 53.85% to 56.36%. In contrast, higher susceptibility was observed for aminoglycosides and carbapenems. Resistance was more frequent in ESBL-producing strains, with limited presence of MDR strains. Age and sex were associated with an increased risk of resistance.

**Conclusion:**

The *Klebsiella pneumoniae* isolates in this study demonstrated significant resistance to commonly prescribed antibiotics. ESBL production substantially contributed to resistance patterns. Although aminoglycosides and carbapenems have shown relative effectiveness, emerging resistance trends underscore the need for continuous monitoring and strict antibiotic stewardship in hospital settings.

**Clinical trial registration:**

Not applicable.

## Introduction

Antimicrobial resistance, if not addressed, is projected to cause up to 10 million deaths annually and cost the global economy $100 trillion by 2050 [1]. The growing prevalence of multidrug-resistant (MDR) *K. pneumoniae* adds further strain on healthcare systems, particularly in intensive care settings [2, 3]. Resistance to aminoglycosides, quinolones, and β-lactams is widely documented. Although polymyxins and tigecycline remain last-resort options, resistance to these agents is emerging [4].

*Klebsiella pneumoniae* is a gram-negative, encapsulated, non-motile bacterium that belongs to the Enterobacteriaceae family. It is recognized as one of the top ten pathogens responsible for hospital-acquired infections, including urinary tract infections, pneumonia, and soft tissue infections. Treatment typically involves third- or fourth-generation cephalosporins for community-acquired infections and carbapenems for hospital-acquired cases [5]. The virulence of *K. pneumoniae* is enhanced by several structural and molecular factors, including the polysaccharide capsule, lipopolysaccharide outer membrane, fimbriae, and siderophores. These features enable the rapid spread and development of resistance. In healthcare settings, *K. pneumoniae can be transmitted* through the gastrointestinal tract of patients or through the hands of medical staff, contributing to nosocomial outbreaks. Of particular concern is the rise of extended-spectrum β-lactamase (ESBL)-producing strains, which significantly reduce the available treatment options [6, 7].

Moreover, *K. pneumoniae* serves as a major reservoir and transmission vehicle for antimicrobial-resistance genes. It plays a critical role in horizontal gene transfer and global dissemination of resistance mechanisms across bacterial species, making it a key focus in public health surveillance [8].

The global spread of carbapenem-resistant *K. pneumoniae* (CRKP) has become an urgent health threat, prompting the World Health Organization to classify it as a critical priority pathogen [2]. According to the CDC, approximately 80% of carbapenem resistance in healthcare settings is linked to *K. pneumoniae* [5].

In Saudi Arabia, the burden of antimicrobial resistance (AMR) continues to rise, particularly among gram-negative pathogens such as *Klebsiella pneumoniae*.

Despite national efforts, local epidemiological data remain limited. Local surveillance is essential to monitor these patterns and inform clinical decision-making [4]. Nevertheless, there remains a lack of detailed region-specific data on resistance trends in *K. pneumoniae* in Saudi Arabia, particularly in the Al-Qassim region [9]. This study addresses that gap by analyzing resistance patterns from a large sample of clinical isolates in Buraydah, providing a focused regional perspective within the broader Gulf healthcare context. This study aimed to evaluate the antibiotic resistance patterns of *K. pneumoniae isolates collected from* the King Fahad Specialist Hospital in Buraydah between 2015 and 2023.

## Objectives

### General objective

To evaluate the antibiotic resistance patterns and susceptibility profiles of *Klebsiella pneumoniae* isolates collected from King Fahad Specialist Hospital in Buraydah, Saudi Arabia between January 2015 and December 2023.

### Specific objectives

- To analyze the distribution of *K. pneumoniae* infections according to patient demographics, including age and sex.
- To assess the variation in resistance patterns across different clinical specimen types.
- To compare antibiotic resistance rates over time across the study period.
- To examine the association between ESBL production and factors such as age, sex, and hospital department.
- To identify departments or clinical settings with the highest rates of resistant infections.

## Methodology

### Study design

This retrospective cross-sectional study was conducted at King Fahad Specialist Hospital (KFHS) in Buraydah, Saudi Arabia. Electronic medical records were reviewed for all eligible patients between January 2015 and December 2023. King Fahad Specialist Hospital is the largest tertiary care hospital in the Qassim region. It receives referrals from all cities and governorates in the region, not just Buraydah, which ensures that the isolates collected are representative of the broader population across the Qassim province.

### Study population and sample size

All patients with confirmed *Klebsiella pneumoniae* infections based on the Clinical and Laboratory Standards Institute (CLSI) criteria were included [10]. The analysis was limited to cases for which antibiotic susceptibility data were available. In total, 3,838 unique patient records were included. Emergency department encounters (*n* = 342) were identified in the source database but excluded from the analytic cohort, because they represented outpatient (OPD) visits rather than the inpatient/clinic-based population under study.

### Inclusion criteria

- Patients of any age and gender diagnosed with *K. pneumoniae* infection.
- Confirmed susceptibility testing results available in the hospital database.

### Exclusion criteria

- Records lacking antibiotic susceptibility testing data.

### Ethical approval

The study was approved by the Qassim Regional Research Ethics Committee, General Directorate of Health Affairs (Approval No. 607/45/3771; Approval Date: October 4, 2023). Patient data were anonymized to ensure confidentiality.

### Definitions

- ESBL: Isolates producing extended-spectrum β-lactamase enzymes.
- ESBL-MDR: ESBL-producing isolates that additionally met multidrug-resistance (MDR) criteria, defined as acquired non-susceptibility to at least one agent in three or more antimicrobial categories, per Magiorakos et al.
- Non-MDR isolates: Isolates that did not meet the ESBL-producing or MDR criteria above. This group includes isolates resistant to one or two antimicrobial classes, as well as isolates fully susceptible to all antimicrobials tested; it does not itself imply a specific level of resistance.
- Intermediate: Isolates with reduced susceptibility based on CLSI breakpoints [10].

### Microbiological identification and antimicrobial susceptibility testing

Bacterial identification and antimicrobial susceptibility testing were performed using two automated microbiology systems available at the hospital laboratory: BD Phoenix and Beckman Coulter MicroScan. Antimicrobial susceptibility was determined by minimum inhibitory concentration (MIC), generated automatically by these systems, and interpreted according to CLSI breakpoints current at the time of testing. Quality control strains and CLSI document version by year were not centrally recorded and are noted as a limitation.

### Data collection

Demographic and clinical data, including patient age, sex, type of clinical specimen, hospital department, and antimicrobial susceptibility profiles, were extracted.

### Data analysis

Data were analyzed using R software (R Foundation for Statistical Computing, Vienna, Austria) and Python (pandas, numpy, scipy). Descriptive statistics were used to summarize the demographic and clinical variables. Chi-square and Fisher’s exact tests were used to assess associations between categorical variables. Descriptive statistics, chi-square/Fisher’s exact tests, and the age-and-sex logistic regression model were performed in R. The multivariable logistic regression, temporal trend analysis, and department-level resistance rates were performed in Python. Logistic regression modeling was used to identify the predictors of ESBL resistance. Visualization techniques, such as bar charts and probability plots, were generated to support interpretation.

Although the study was originally designed to cover the period 2013–2023, isolates from 2013 to 2014 were recorded only in the hospital’s non-digitized (paper-based) laboratory registers prior to its transition to an electronic laboratory information system in 2015, and could not be systematically extracted for this analysis. Consequently, all 3,838 isolates included in this study, and the temporal trend analysis described below, reflect the period 2015–2023; isolates were grouped into three consecutive intervals (2015–2017, 2018–2020, and 2021–2023) to improve the stability and interpretability of resistance estimates given the smaller annual sample sizes in the earliest years of this range.

## Results

### Demographic and clinical characteristics

A total of 3,838 confirmed *K. pneumoniae* infections were included. Among them, 2,202 (57.4%) were female and 1,636 (42.6%) were male. The mean age of the patients was 71 years for females and 63 years for males. The age range for female patients ranged from 4 to 87 years, while for males, it ranged from 32 to 94 years (Table 1).

**Table 1:** Distribution of age by sex

Female	Male
2202	1636
Mean	Mean
71	63
Oldest – Youngest	Oldest – Youngest
87 − 4	94 − 32

### Bivariate associations (chi-square and fisher’s exact tests)

Chi-square and Fisher’s exact tests were used to assess associations between key categorical variables, as referenced in the Methods. Results are summarized in Table 1a.

**Table 1a:** Bivariate association test results. All tested associations were statistically significant, consistent with the department-, specimen-, and antibiotic-level differences described throughout the results

Variable 1	Variable 2	Test statistic	df	*p*-value
Gender	Antibiotic	χ² = 236.25	39	< 2.2 × 10⁻¹⁶
Antibiotic	Result	Fisher’s exact (simulated)	—	0.0005
Result	Resistance category	χ² = 487.67	4	< 2.2 × 10⁻¹⁶
Department	Resistance category	Fisher’s exact (simulated)	—	0.0005
Specimen type	Resistance category	χ² = 351.37	8	< 2.2 × 10⁻¹⁶

### Distribution by specimen type

Blood was the most common specimen source (2,540 cases, 66.18%), followed by urine (676, 17.61%), body fluids (555, 14.46%), and tissue samples (67, 1.75%) (Table 2).

**Table 2:** Distribution of clinical specimen types

Specimen Type	Sample Count
BODY FLUID	555
TISSUE	67
URINE	676
Blood culture	2540

### Distribution by clinical department

The majority of the isolates were recovered from the ICU (*n* = 624), SICU (*n* = 621), and trauma room (*n* = 504). In females, the trauma room had the highest number of cases (*n* = 504), while in males, the SICU recorded the most (*n* = 621). The lowest numbers were reported in nephrology and hemodialysis clinics (Table 3).

**Table 3:** Distribution of cases by clinical department

Clinics	Female	Male	Grand Total
DAY SURGERY UNIT	24		24
FEMALE MEDICAL WARD	364		364
GENERAL FEMALE SURGERY WARD	270		270
Hemodialysis Clinic -	18		18
HOME Clinics		36	36
ICU	262	362	624
Lab. Reception	34		34
MALE EENT WARD		286	286
Male Surgery Ward	15	87	102
Medical Ward		32	32
NEPHROLOGY CLINIC	15		15
Peritoneal Dialysis Clinic	274	54	328
PNEUMOLOGY CLINIC	160		160
Podiatric Clinic		14	14
Rehabilitation Medicine	64		64
SICU		621	621
Stroke Unit	120		120
Trauma Room	504		504
UROLOGY	78	144	222
Grand Total	**2202**	**1636**	**3838**

### Resistance classification

The resistance categories were defined as ESBL, ESBL-MDR, and Non-MDR isolates (resistance to 1–2 antibiotics). ESBL isolates (*n* = 877) had the highest number of resistant cases, followed by Non-MDR isolates (*n* = 833). Intermediate resistance was more common in the Non-MDR group (*n* = 102) than that in the ESBL group (*n* = 50). ESBL-MDR showed the lowest counts in all categories (Table 4).

**Table 4:** Distribution of resistance categories

	ESBL	ESBL-MDR	Non-MDR
Intermediate	50	18	102
Resistant	877	84	833
Sensitive	352	18	1504

*ESBL: extended-spectrum beta-lactam, ESBL-MDR: extended-spectrum beta-lactamase with multidrug resistance (≥ 3 antimicrobial classes); Non-MDR: isolates not meeting ESBL or MDR criteria (includes isolates resistant to one or two classes and fully susceptible isolates)

### Resistance by department

Analysis by department revealed that resistance was highest in the trauma room (*n* = 392), SICU (*n* = 324), and ENT ward (*n* = 264). The ICU had the highest number of sensitive cases (*n* = 526) followed by the SICU (*n* = 243). Intermediate resistance was most common in the SICU (*n* = 54) (Table 5).

**Table 5:** Distribution of resistance by clinical department

Row Labels	Intermediate	Resistant	Sensitive	Grand Total
DAY SURGERY UNIT	4 (16.67%)	11 (45.83%)	9 (37.5%)	24
FEMALE MEDICAL WARD	13 (3.57%)	159 (43.68%)	192 (52.75%)	364
GENERAL FEMALE SURGERY WARD	8 (2.96%)	103 (38.15%)	159 (58.89%)	270
Hemodialysis Clinic	2 (11.11%)	1 (5.56%)	15 (83.33%)	18
HOME Clinics	6 (16.67%)	4 (11.11%)	26 (72.22%)	36
ICU	15 (2.4%)	83 (13.3%)	526 (84.29%)	624
Lab. Reception			34 (100.0%)	34
MALE EENT WARD		264 (92.31%)	22 (7.69%)	286
Male Surgery Ward	3 (2.94%)	48 (47.06%)	51 (50.0%)	102
Medical Ward		2 (6.25%)	30 (93.75%)	32
NEPHROLOGY CLINIC			15 (100.0%)	15
Peritoneal Dialysis Clinic	30 (9.15%)	103 (31.4%)	195 (59.45%)	328
PNEUMOLOGY CLINIC		80 (50.0%)	80 (50.0%)	160
Podiatric Clinic		1 (7.14%)	13 (92.86%)	14
Rehabilitation Medicine			64 (100.0%)	64
SICU	54 (8.7%)	324 (52.17%)	243 (39.13%)	621
Stroke Unit	18 (15.0%)	84 (70.0%)	18 (15.0%)	120
Trauma Room		392 (77.78%)	112 (22.22%)	504
UROLOGY	17 (7.66%)	135 (60.81%)	70 (31.53%)	222
Grand Total	170 (4.43%)	1794 (46.74%)	1874 (48.83%)	3838

### Antibiotic resistance rates

Resistance to cefotaxime was 68.69%, followed by ceftazidime (59.18%), amoxicillin-clavulanate (58.38%), ciprofloxacin (56.36%), levofloxacin (53.85%), and piperacillin-tazobactam (52.81%). On the other hand, amikacin (19.63%), gentamicin (34.09%), imipenem (33.06%), and ertapenem (34.96%) showed lower resistance rates. These values are summarized in Table 6.

**Table 6:** Resistance rates of *Klebsiella pneumoniae* isolates to selected antibiotics

Antibiotic	Resistance (%)
Cefotaxime (*n* = 214)	68.69%
Ceftazidime (*n* = 245)	59.18%
Amoxicillin-Clavulanate (*n* = 197)	58.38%
Ciprofloxacin (*n* = 220)	56.36%
Levofloxacin (*n* = 208)	53.85%
Piperacillin-Tazobactam (*n* = 231)	52.81%
Amikacin (*n* = 219)	19.63%
Gentamicin (*n* = 220)	34.09%
Imipenem (*n* = 245)	33.06%
Ertapenem (*n* = 123)	34.96%

### Temporal trend in antibiotic resistance (2015–2023)

To address the objective of comparing resistance rates over time, isolates from the 2015–2023 analysis window (see Methods) were grouped into three sequential periods: 2015–2017, 2018–2020, and 2021–2023. Overall resistance (pooled across all tested antibiotics) declined from 62.50% (2015–2017) to 48.90% (2018–2020) and 43.59% (2021–2023), suggesting a gradual downward trend in resistance over the study period, although annual-level estimates were unstable due to small early-year sample sizes and could not be reliably modeled.

**Table:** 

Period	*n*	% Resistant
2015–2017	272	62.50%
2018–2020	1313	48.90%
2021–2023	2253	43.59%

### Multivariable predictors of ESBL production

A multivariable logistic regression model was constructed to identify independent predictors of ESBL production, including age, sex, specimen type, and hospital department (reference categories: Blood culture for specimen type; pooled remaining departments for department). Trauma Room and SICU were excluded from this model because ESBL status showed complete separation in these departments (100% and 0% of isolates, respectively), which precluded stable odds ratio estimation; these two departments are reported descriptively only. Antibiotic name was not included as a predictor because it was collinear with the outcome definition itself.

**Table:** 

Variable	OR	95% CI	*p*-value
Age (per year)	1.012	1.004–1.020	0.004
Sex: Male (ref. Female)	0.968	0.721–1.299	0.827
Specimen: Body fluid (ref. Blood culture)	0.377	0.264–0.539	< 0.001
Specimen: Tissue (ref. Blood culture)	15.601	7.917–30.741	< 0.001
Specimen: Urine (ref. Blood culture)	1.443	1.080–1.927	0.013
Department: Female Medical Ward (ref. Other)	12.872	6.274–26.405	< 0.001
Department: ICU (ref. Other)	0.097	0.056–0.169	< 0.001
Department: Peritoneal Dialysis Clinic (ref. Other)	14.299	10.230–19.987	< 0.001

Descriptively, ESBL production was 100% (504/504) in the Trauma Room and 0% (0/621) in the SICU across the study period; this all-or-none pattern is not biologically plausible for a single department over a 9-year span and may reflect a department-level data-recording artifact rather than a confirmed true clinical effect; this could not be verified against source laboratory records within the scope of this study. This pattern, and its implication for the reliability of department-level resistance comparisons, is discussed further in the Limitations section.

### Antibiotic activity patterns

**Fig. 1:**
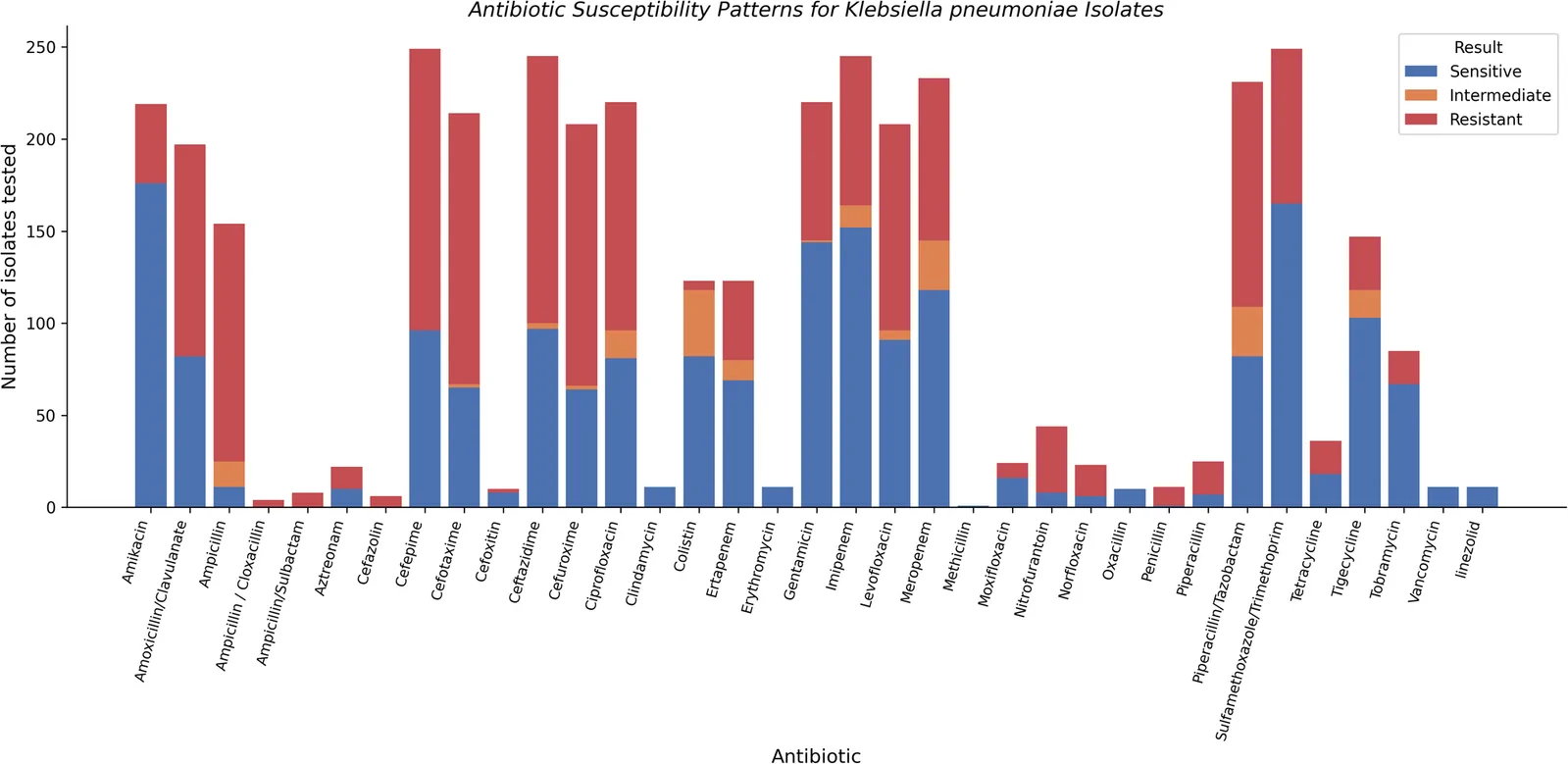
Antibiotic susceptibility patterns for *K. pneumoniae*

Figure 1 shows the antibiotic activity patterns against *K. pneumoniae* from 2015 to 2023. Piperacillin/tazobactam and amoxicillin/clavulanic acid are among the most frequently tested antibiotics. Amoxicillin/clavulanic acid showed high intermediate resistance (~ 250 cases), while piperacillin/tazobactam had nearly equal intermediate and resistant responses (~ 150 each). Colistin and ertapenem were the least tested but showed a higher resistance rate relative to their total counts.

### ESBL prediction by age and gender

**Fig. 2:**
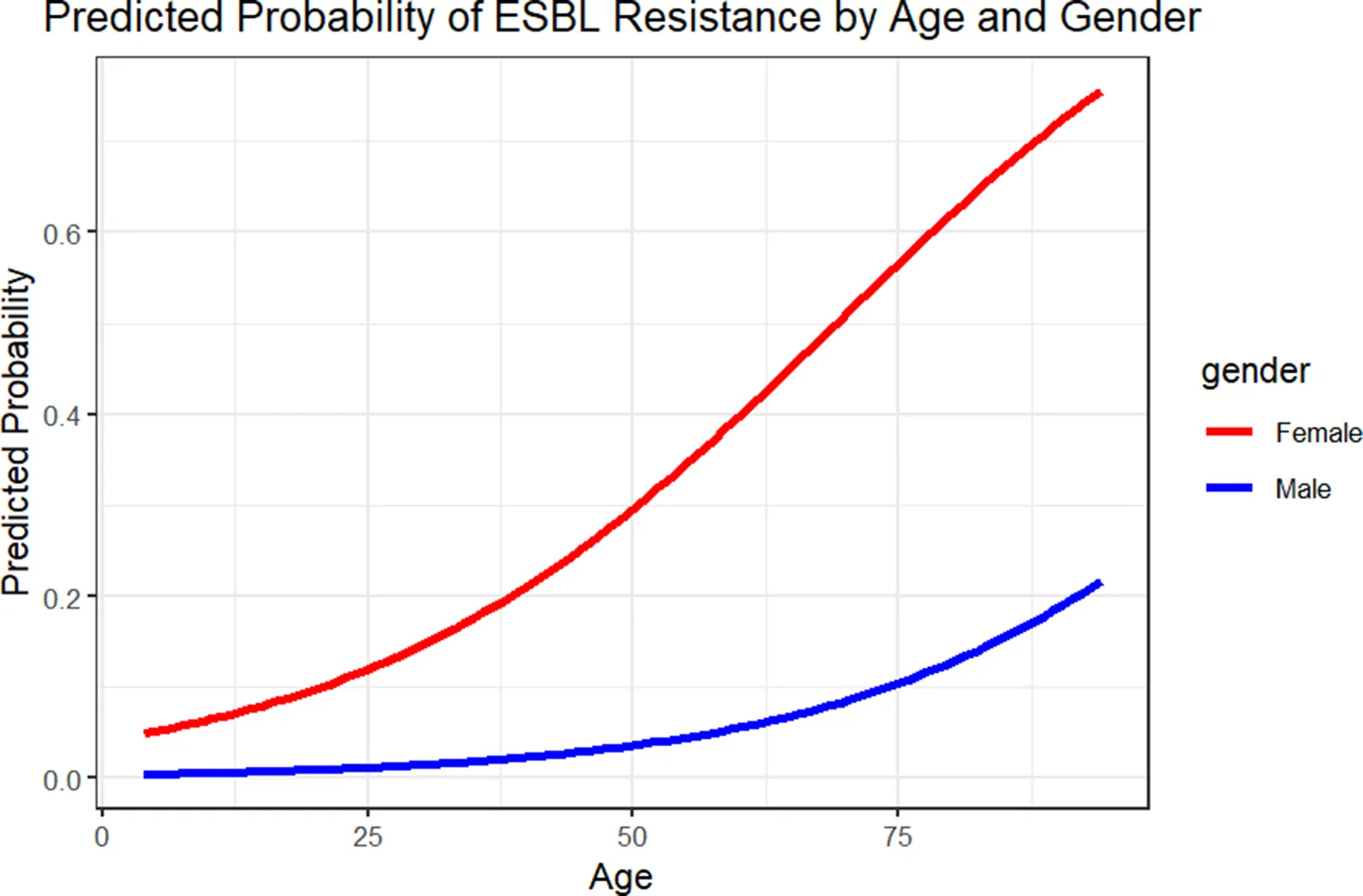
Predicted probability of ESBL production according to age and sex

Figure 2 shows the nonlinear relationship between ESBL resistance and age. The probability of ESBL resistance increases with age, particularly in female patients. An interaction effect between age and sex on resistance risk was observed, suggesting a demographic influence on resistance patterns.

## Discussion

The present study examined the antibiotic resistance patterns of *Klebsiella pneumoniae* isolates collected from a major tertiary hospital in Buraydah, Saudi Arabia. These findings indicate a high prevalence of resistance to first-line antibiotics, particularly third-generation cephalosporins and fluoroquinolones.

Resistance to cefotaxime (68.69%) and ceftazidime (59.18%) was consistent with previous reports from the same hospital, where rates of 66.9% and 77%, respectively, were observed [7]. Similarly, fluoroquinolone resistance was substantial, with 56.36% ciprofloxacin and 53.85% levofloxacin, consistent with earlier findings in Al-Madinah (57.7% and 61.1%, respectively) [7]. However, other studies have reported lower resistance rates to fluoroquinolones, such as 26.8% for levofloxacin and 35.7% for ciprofloxacin [11], indicating regional variation in susceptibility patterns.

Amikacin (80.37%) and gentamicin (65.45%) showed high susceptibility rates in this study, consistent with Ministry of Health reports recommending aminoglycosides as part of combination therapy [12]. Carbapenems, including imipenem (62.04%) and ertapenem (56.10%), also retained relatively good activity, although the emergence of resistant strains remains a concern. A cross-sectional study from the southern region of Saudi Arabia reported carbapenem resistance in 57.4% of *K. pneumoniae* isolates, indicating ongoing challenges in treatment [13].

Our data showed that ESBL-producing strains accounted for the most resistant cases, whereas a smaller proportion of isolates were classified as MDR. This pattern reflects global trends in *K. pneumoniae* resistance [4, 8]. In addition, demographic variables such as age and sex were significantly associated with resistance, with older female patients showing a higher likelihood of ESBL positivity (Fig. 2).

The department-level analysis revealed that the trauma room and SICU had the highest rates of resistance. This is consistent with prior studies that linked high antimicrobial resistance to critical care units [14]. The misuse of antibiotics in clinical settings, as reported in previous literature (with misuse rates ranging from 41% to 92% in Saudi Arabia), may contribute to this trend [14].

Although piperacillin-tazobactam is frequently recommended as an empirical therapy, our findings showed a resistance rate of 52.81% and intermediate responses were also common. These results raise concerns regarding its continued effectiveness and highlight the importance of tailored empirical therapy based on local resistance profiles.

This study also highlights the urgent need for surveillance programs and stewardship interventions. Because *K. pneumoniae* remains a key driver of nosocomial infections, understanding its resistance trends is vital for optimizing treatment and infection control strategies.

These findings are directly relevant to ongoing national and global efforts to contain antimicrobial resistance. Saudi Arabia’s Ministry of Health has pursued antimicrobial stewardship and infection control as part of the Vision 2030 Health Sector Transformation Program [15], alongside the Kingdom’s National Action Plan on Combating Antimicrobial Resistance (first issued in 2017 and updated for 2022–2025) [16], which is aligned with the WHO Global Action Plan on AMR and includes strengthening AMR surveillance and laboratory capacity as core objectives. The declining resistance trend observed across our three study periods (2015–2017 to 2021–2023) is encouraging in this context, while the continued high burden of ESBL-producing and carbapenem-resistant isolates highlights the importance of sustaining and further strengthening surveillance and antimicrobial stewardship efforts in the region to build on the progress already achieved. This work also contributes regional evidence toward the United Nations Sustainable Development Goals, particularly Goal 3 (Good Health and Well-being), which identifies antimicrobial resistance as a threat to global health security requiring strengthened national surveillance capacity.

A key strength of this study lies in the large dataset collected from the region’s main referral hospital. While this enhances the reliability of the findings, generalizability may be limited, as data from other healthcare institutions were not included due to technical constraints. The study contributes valuable, up-to-date resistance data that support the broader goals of national antimicrobial surveillance in Saudi Arabia. The high prevalence of multidrug-resistant Klebsiella pneumoniae observed reinforces the need to optimize empirical therapy and strengthen infection control protocols. Because pre-2015 laboratory records at this hospital were paper-based and could not be systematically digitized for this analysis, the present results offer a critical baseline for future investigations and national-level monitoring.

## Limitations

Several limitations should be considered when interpreting these findings:

- This was a retrospective, single-center study conducted at one tertiary hospital, which may limit generalizability to other regions or healthcare settings.
- Molecular characterization of resistance mechanisms (e.g., blaCTX-M and other ESBL genes, carbapenemase genes such as KPC, NDM, or OXA-48) was not performed; resistance categories were based on phenotypic susceptibility results only.
- Clonality analysis (e.g., sequence typing) was not conducted, so it is not possible to distinguish clonal spread from independent acquisition of resistance.
- Clinical variables that could confound resistance patterns, including prior antibiotic exposure, comorbidities, and healthcare-associated versus community-acquired classification, were not available in the dataset.
- Patient outcome data (e.g., mortality, length of stay) were not available and could not be linked to resistance status.
- CLSI breakpoints were revised multiple times over the 2015–2023 study period; changes in interpretive criteria over time may partially explain apparent temporal trends in resistance and could not be fully disentangled from true biological change.
- Quality control strain records and the specific CLSI document edition in use for each year of the study period were not centrally archived by the laboratory, so year-to-year consistency in interpretive criteria could not be independently verified.
- Emergency department encounters (*n* = 342) were excluded because they represented a distinct outpatient population; resistance patterns in emergency department isolates were not assessed and may differ from the inpatient/clinic-based cohort reported here.
- Several departments (notably Trauma Room and SICU) showed complete separation in ESBL classification (100% and 0% of isolates, respectively) across the study period. This pattern is not biologically plausible and may reflect a department-level recording artifact rather than true clinical variation, though this could not be independently confirmed; these two departments were therefore excluded from the multivariable regression model and should be interpreted with caution in any department-level comparison.
- Department and calendar-year period were highly collinear in this dataset (several departments contributed cases in only one time period), which prevented department and period from being modeled jointly; temporal trends and department-level associations with ESBL are therefore reported as separate analyses rather than a single combined model.
- Antibiotic names were recorded with inconsistent spelling and abbreviations in the source data (e.g., “Gentamicin” vs. “Gentamycin”); these were consolidated prior to analysis, but residual misclassification cannot be entirely excluded.

## Conclusion

The findings of this study demonstrate a high prevalence of antibiotic resistance among *Klebsiella pneumoniae* isolates in a tertiary hospital setting in Buraydah, Saudi Arabia. Resistance to commonly used antimicrobials, including cephalosporins, fluoroquinolones, and beta-lactam/beta-lactamase inhibitor combinations, was particularly elevated.

Aminoglycosides and carbapenems retained relatively high activity, although resistance trends in some departments raised concerns about their continued efficacy. The widespread presence of ESBL-producing strains along with a smaller subset of MDR cases underscores the complexity of treating *K. pneumoniae* infections in clinical practice.

These results highlight the urgent need for:

- Institutional antimicrobial stewardship programs.
- Continuous surveillance of resistance patterns.
- Strict adherence to infection control protocols.

Regional data, such as those used in this study, are essential for informing empirical treatment guidelines and shaping national health policies to address antimicrobial resistance threats.

## Data Availability

The data supporting the findings of this study are available from the corresponding author, Prof. Noorah Saleh Al-Sowayan, upon reasonable request.

## Abbreviations

AMR: Antimicrobial Resistance
MDR: Multidrug Resistance
*K. pneumoniae*: *Klebsiella pneumoniae*
AST: Antimicrobial Susceptibility Testing
